# RETRACTION: Effect of Incisional Negative Pressure Therapy and Conventional Treatment on Wound Complications After Orthopaedic Trauma Surgery: A Meta‐Analysis of Randomized Controlled Studies

**DOI:** 10.1111/iwj.70380

**Published:** 2025-03-21

## Abstract

**RETRACTION:** LiP. and LiJ. , “Effect of Incisional Negative Pressure Therapy and Conventional Treatment on Wound Complications after Orthopaedic Trauma Surgery: A Meta‐Analysis of Randomized Controlled Studies,” International Wound Journal 20, no. 10 (2023): 4291–4299, 10.1111/iwj.14331.37534409 PMC10681432

The above article, published online on 03 August 2023, in Wiley Online Library (http://onlinelibrary.wiley.com/), has been retracted by agreement between the journal Editor in Chief, Professor Keith Harding; and John Wiley & Sons Ltd. Following an investigation by the publisher, all parties have concluded that this article was accepted solely on the basis of a compromised peer review process. The editors have therefore decided to retract the article. The authors did not respond to our notice regarding the retraction.

**RETRACTION:** P. Li and J. Li , “Effect of Incisional Negative Pressure Therapy and Conventional Treatment on Wound Complications after Orthopaedic Trauma Surgery: A Meta‐Analysis of Randomized Controlled Studies,” International Wound Journal 20, no. 10 (2023): 4291–4299, 10.1111/iwj.14331.37534409 PMC10681432

The above article, published online on 03 August 2023, in Wiley Online Library (http://onlinelibrary.wiley.com/), has been retracted by agreement between the journal Editor in Chief, Professor Keith Harding; and John Wiley & Sons Ltd. Following an investigation by the publisher, all parties have concluded that this article was accepted solely on the basis of a compromised peer review process. The editors have therefore decided to retract the article. The authors did not respond to our notice regarding the retraction.

